# Genome-Wide Analysis of Long Non-coding RNAs Involved in Nodule Senescence in *Medicago truncatula*

**DOI:** 10.3389/fpls.2022.917840

**Published:** 2022-05-30

**Authors:** Lin Yu, Tengda Huang, Xinyu Qi, Jingsu Yu, Tian Wu, Zupeng Luo, Lei Zhou, Yixing Li

**Affiliations:** State Key Laboratory for Conservation and Utilization of Subtropical Agro-Bioresources, College of Animal Science and Technology, Guangxi University, Nanning, China

**Keywords:** long non-coding RNA, *Medicago truncatula*, nodule, senescence, RNA-Seq

## Abstract

Plant long non-coding RNAs (lncRNAs) are widely accepted to play crucial roles during diverse biological processes. In recent years, thousands of lncRNAs related to the establishment of symbiosis, root nodule organogenesis and nodule development have been identified in legumes. However, lncRNAs involved in nodule senescence have not been reported. In this study, senescence-related lncRNAs were investigated in *Medicago truncatula* nodules by high-throughput strand-specific RNA-seq. A total of 4576 lncRNAs and 126 differentially expressed lncRNAs (DElncRNAs) were identified. We found that more than 60% lncRNAs were associated with transposable elements, especially TIR/Mutator and Helitron DNA transposons families. In addition, 49 DElncRNAs were predicted to be the targets of micro RNAs. Functional analysis showed that the largest sub-set of differently expressed target genes of DElncRNAs were associated with the membrane component. Of these, nearly half genes were related to material transport, suggesting that an important function of DElncRNAs during nodule senescence is the regulation of substance transport across membranes. Our findings will be helpful for understanding the functions of lncRNAs in nodule senescence and provide candidate lncRNAs for further research.

## Introduction

Non-coding RNAs with the length greater than 200 nt are known as long non-coding RNAs (lncRNAs). A large number of lncRNAs have been identified in the genomes of some plant species such as Arabidopsis (Liu et al., 2012), rice (Wang H. et al., 2015), maize (Li et al., 2014), cotton (Wang M. et al., 2015), tomato (Wang et al., 2016), peanut (Ma et al., 2020), *Medicago truncatula* (Wang T. Z. et al., 2017) and soybean (Golicz et al., 2018b). According to their origins in genome, lncRNAs can be classified into sense, antisense, intronic and large intergenic non-coding (linc) types (Rai et al., 2019). Plant lncRNAs play crucial regulatory roles in various biological processes including root development (Chen et al., 2018), flowering (Csorba et al., 2014), seedling photomorphogenesis (Wang et al., 2014), fruit ripening (Zhu et al., 2015), sexual reproduction (Golicz et al., 2018a) and defense responses to biotic (Cui et al., 2020) and abiotic stresses (Wang T. Z. et al., 2017).

Root nodules are special organs formed by legume-rhizobium symbiosis. Emerging evidence suggests that lncRNAs function as crucial regulators of symbiotic nitrogen fixation (SNF) in nodules. A well-known lncRNA associated with SNF is ENOD40 in *M. truncatula* (Campalans et al., 2004) which can act as a dual RNA (Bardou et al., 2011) in nodule organogenesis. Another lncRNA is TAS3 RNA in *M. truncatula* and the miR390/TAS3 pathway plays negative roles in nodulation and nodule development (Hobecker et al., 2017). Recently, thousands of lncRNAs in *M. truncatula* have been identified to be involved in SNF and possibly regulate mRNA expression in *cis* way (Pecrix et al., 2018).

SNF by nodules lasts for a peroid, peaks at some time in the nodule life-span and declines with the senescence of nodules. Mature indeterminate nodules (such as nodules on *M. trunctula* roots) are divided into four developmental regions namely the apical meristematic, the infection, the nitrogen fixation and the senescence zones (Van de Velde et al., 2006). Nodule senescence is a developmental process which is initiated in the senescence zone and advances gradually to the meristematic zone. Although a large number of lncRNAs involved in SNF have been identified, little is known about the lncRNAs related to nodule senescence. Interestingly, several recent reports have suggested that lncRNAs play key roles in leaf senescence (Chao et al., 2019; Huang et al., 2021) and nodule senescence has a relatively high similarity with leaf senescence at the molecular level (Van de Velde et al., 2006), indicating that lncRNAs are also likely to be important regulators during nodule senescence. However, previous work focused on the identification and functions of multiple genes involved in nodule senescence, the research on ageing-related lncRNAs in root nodules has been lacking. In this study, we conducted high-throughput strand-specific RNA-seq of nodules at 21- and 35-days post inoculation (dpi) with *Sinorhizobium meliloti* 1021 to investigate and characterize lncRNAs associated with nodule senescence. Our findings will provide new insights into the underlying functions of lncRNAs during nodule senescence.

## Materials and Methods

### Plant Materials

*M. truncatula* A17 seeds were surface-sterilized in 75% ethanol for 5 min and 2% sodium hypochlorite solution for 15 min, before washing 5–6 times with sterile water. The seeds were placed on 1.5% (w/v) agar plates in 4°C for 1 day. After germination in a greenhouse (20°C/25°C and 16 h/8 h light/dark) for 1–2 days, the seedlings were planted in sterilized sand and watered with Fahraeus nitrogen-free nutrient solution (Fahraeus, 1957). *S. meliloti* 1021 was inoculated after the cotyledons were expanded. Nodules were collected from the taproots of 40 plants at each dpi.

Paraffin sections of nodules were carried out for microscopic observation. Nodules at different dpi were cut longitudinally and fixed with FAA more than 24 h. After dehydrated with gradient ethanol and cleared with dimethylbenzene, the nodules were embedded in paraffin and made into sections. Then the slides were stained with toluidine blue and observed with the Olympus light microscope.

### Library Preparation for Long Non-coding RNA-Seq

Total RNA was obtained using plant RNA extraction kit RN40 (Aidlab, Beijing, China). Nanodrop2000 (Thermo Fisher, Waltham, MA, United States) was employed to verify RNA concentration and purity. Agilent Bioanalyzer 2100 (Agilent Technologies, Santa Clara, CA, United States) was used to verify RNA integrity. Ribo-off rRNA Depletion Kit N409-2 (Vazyme, Nanjing, China) was used to remove rRNA. The VAHTS Total RNA-seq Library Prep Kit for Illumina (Vazyme, Nanjing, China) was used for library construction. The libraries were sequenced on an Illumina NovaSeq 6000 platform (PE150).

### Identification and Analysis of Long Non-coding RNA

Clean data were produced by removing reads containing adapter and low-quality reads from raw data. HISAT2 v2.0.4 (Kim et al., 2019) was used for sequence alignment. The transcriptome was assembled using StringTie v1.3.1 (Pertea et al., 2015) and Scripture based on the reads mapped to the reference *M. truncatula* genome MedtrA17_4.0. The assembled transcripts were compared using the Cuffcompare v2.1.1 program (Trapnell et al., 2010). LncRNAs were screened for using the following criteria: (1) Transcripts less than 200 nt were removed. (2) LncRNA transcripts were evaluated for their potential protein-coding with CPC (Kong et al., 2007), CNCI (Sun et al., 2013), Pfam and CPAT (Wang et al., 2013) platforms, and the intersection of the four methods were retained.

### Identification of Differentially Expressed Long Non-coding RNAs, Target Gene Prediction and Transposable Element Analysis of Long Non-coding RNAs

Differential expression analysis was performed using the DESeq R package v1.10.1 (Anders and Huber, 2010). LncRNAs or mRNAs with *p* < 0.05 and Fold Change ≥ 1.5 were considered to be differentially expressed. For target gene prediction, Perl scripts were used to search adjacent genes within ± 100 kb of lncRNAs as the *cis*-target genes, while *trans*-target gene prediction was based on the complementary sequence using LncTar (Beattie, 2014) prediction program. The target lncRNAs of microRNAs were predicted using TargetFinder (v1.0; Fahlgren and Carrington, 2010). Extensive *de-novo* TE Annotator (EDTA; Ou et al., 2019)^1^ was used for Transposable Element (TE) annotation. The lncRNA overlapping with TE-site but not completely inside a TE was confirmed as TE-associated lncRNA (Wang D. et al., 2017).

### Gene Annotation and Functional Analysis of Differentially Expressed Long Non-coding RNAs Target Genes

Gene function was annotated based on the databases of Nr (NCBI nonredundant protein sequences^2^), Pfam (Protein family^3^), KOG/COG (Clusters of Orthologous Groups of proteins^4^), Swiss-Prot (a manually annotated and reviewed protein sequence database^5^), KEGG (Kyoto Encyclopedia of Genes and Genomes^6^) and GO (Gene Ontology^7^). GO enrichment analysis was implemented by the TopGO R packages, and KOBAS (Mao et al., 2005) software was used for KEGG pathway analysis.

### Quantitative Real-Time PCR

Total RNA was extracted by TRizol regent and random reverse primer was used for reverse transcription of lncRNA and mRNA. The qPCR was performed on qTOWER 3.0 real-time PCR System (Analytik, Jena, Germany). Primers are listed in Supplementary Table 1. The relative expression levels of genes were calculated by 2^–ΔΔCT^ method.

### Statistical Analysis

Statistical analysis was performed using SPSS 19.0 software (IBM, Chicago, IL, United States). Two groups of data were analyzed using the unpaired two-tailed *t*-test. Significance analysis of length and expression level between TE- and non-TE-lncRNAs was performed by Wilcoxon test.

## Results

### Nodules at 35 Days Post Inoculation Displayed Senescence

**FIGURE 1:**
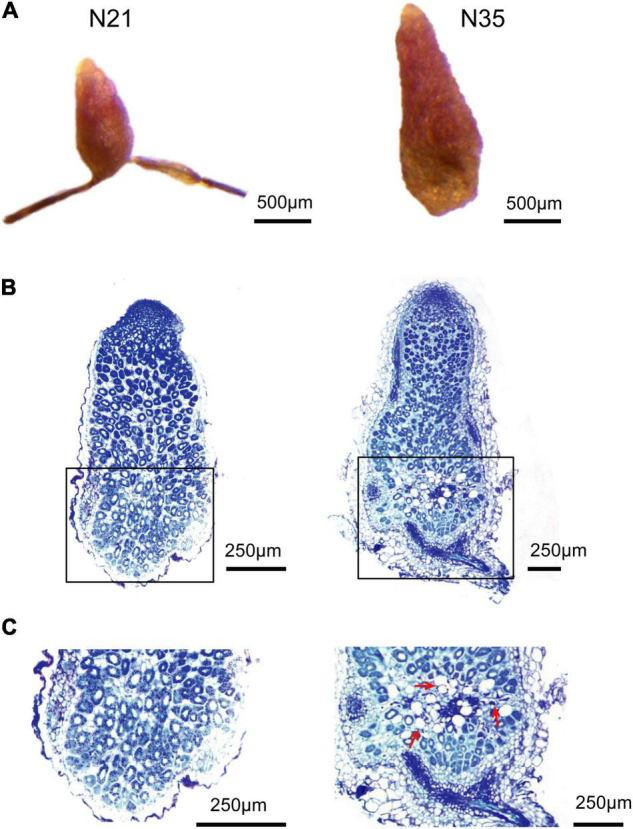
Microscopic analysis of nodule senescence. **(A)** The appearance of N21 and N35. **(B)** Paraffin-embedded slides of N21 and N35 stained with toluidine blue. **(C)** Enlargement of the rectangle in B. N21, N35, nodules at 21 or 35 dpi. Bars = 500 μm **(A)** and 250 μm **(B,C)**. The arrows point to the aging cells.

Nodules at 21 and 35 dpi were collected to determine their developmental stage. At 21 dpi, nodules were small and pink, while at 35 dpi, a small proximal section gained a green color, indicating that aging has occurred (Figure 1A). Paraffin sections stained with toluidine blue were performed to observe the developmental zones. The cells in nitrogen fixation zone of 21 dpi nodules remained healthy with a large number of bacteroids. While at 35 dpi, a small distinct senescence zone was present at the proximal region of the fixation zone (Figure 1B). In this region, the number of infected cells reduced and the loss of cellular content was observed, indicating the degradation of bacteroids (Figure 1C). Moreover, some infected cells were abnormal with a very large vacuole. According to the above observations, we determined that 35 dpi nodules have entered the aging stage.

### Identification and Characterization of Long Non-coding RNAs by High-Throughput RNA-Seq

**FIGURE 2:**
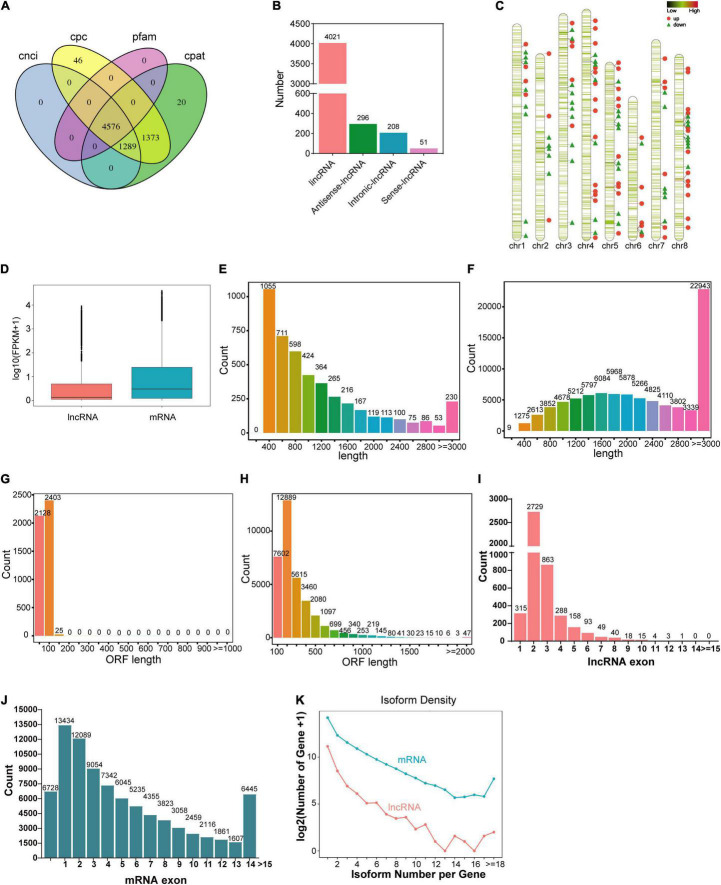
Global identification and characterizations of lncRNAs. **(A)** Prediction of lncRNAs by CPC, CNCI, Pfam, and CPAT. **(B)** The classification of lncRNAs. **(C)** Chromosome distribution of lncRNAs and DElncRNAs. The green horizontal lines represent lncRNAs, the red dots represent up-regulated DElncRNAs and the green triangles represent down-regulated DElncRNAs. **(D)** The expression level of lncRNA and mRNA. **(E,F)** The length of lncRNAs and mRNAs. **(G,H)** The length of ORF for lncRNAs and mRNAs. **(I,J)** Exon number of lncRNAs and mRNAs. **(K)** Isoform number of lncRNAs and mRNAs.

For genome-wide identification of lncRNAs involved in nodule senescence, transcripts were assembled from high-throughput strand-specific RNA-seq of *M. truncatula* nodules harvested at 21 dpi (N21) and 35 dpi (N35) with three biological replicates. A total of 55,596,472 to 71,305,005 clean reads were generated from each sample with a Q30 higher than 92.26% (Supplementary Table 2). The unique map ratio of clean reads to *M. truncatula* genome MedtrA17_4.0 ranged from 66.32 to 74.16% (Supplementary Table 3). In total, 4576 lncRNAs were obtained (Figure 2A). The majority of the lncRNAs belonged to lincRNAs (4021, 87.9%), followed by antisense (296, 6.5%), intronic (208, 4.5%) and sense (51, 1.1%) lncRNAs (Figure 2B). The distribution of lnRNAs in chromosomes revealed an obvious preference for chromosome 8 (8.51 per Mb), 5(8.37 per Mb) and 4(8.01 per Mb; Figure 2C).

We compared the expression level, transcript and ORF length, exon number, and the isoform number of lncRNAs with mRNAs. The results reflected the different characterizations between lncRNAs and mRNAs. The average expression level of mRNAs was 1.8 times that of lncRNAs (Figure 2D). Most lncRNAs have a transcript length of less than 1000 nt (71.1%; Figure 2E) and an ORF length ≤ 100aa (Figure 2G). In contrast, the average length of mRNAs was 2467nt (Figure 2F) and the ORF of most mRNAs was longer than 100aa (Figure 2H). The majority of lncRNAs contained less than three exons (Figure 2I), while about 76.5% mRNAs have more than three exons (Figure 2J). For isoform number, the presence of one or two isoforms is the most common case for both lncRNAs and mRNAs (Figure 2K).

### Identification and Functional Analysis of Long Non-coding RNAs Related to Nodule Senescence

**FIGURE 3:**
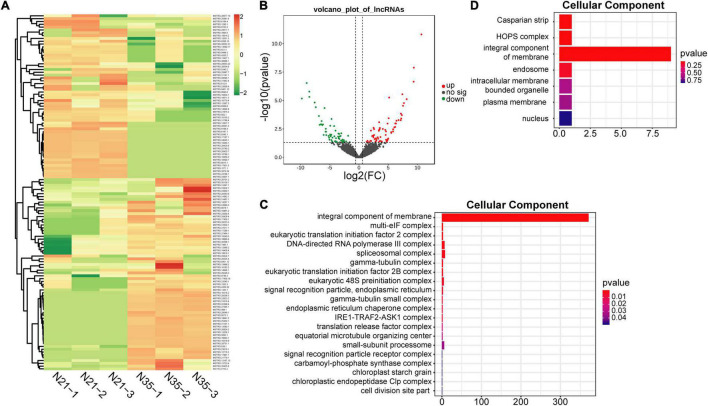
Identification and functional analysis of DElncRNAs during nodule senescence. **(A)** Heat maps showed the fold changes of DElncRNAs. N21-1, N21-2, N21-3 and N35-1, N35-2, N35-3 represent three biological replicates of N21 and N35. **(B)** Volcano map of DElncRNAs. **(C,D)** GO analysis of *cis* and *trans* target genes of DElncRNAs. Top 20 terms of cellular component were displayed.

A total of 126 DElncRNAs including 67 up-regulated and 59 down-regulated lncRNAs were identified (N35 vs N21; Figures 3A,B). The distribution of DElnRNAs in chromosomes displayed the same preference with total lncRNAs. Moreover, although chromosome 6 had a small number of DElncRNAs, most of them were up-regulated (Figure 2C). Many lncRNAs function by regulating gene expression, so the prediction of their target genes can provide insight into their biological roles. A total of 1911 putative *cis*-regulated and 28 *trans*-regulated target genes of DElncRNAs were predicted. GO terms analysis of these target genes showed significant differences between the mature and senescent nodules. Notably, for the top 20 terms of cellular component, integral component of membrane was the most significantly enriched term by both *cis* (Figure 3C) and *trans* (Figure 3D) target genes. KEGG pathway analysis revealed that *cis* target genes were enriched in RNA polymerase, purine and pyrimidine metabolism, as well as flavonoid and amino acids biosynthesis pathways (Supplementary Figure 1). The *trans* target genes were enriched in MAPK signaling, plant hormone signal transduction, plant-pathogen interaction and isoflavonoid biosynthesis pathways (Supplementary Figure 1).

### Identification of Long Non-coding RNAs Targeting Memebrane-Related Genes and Transcription Factors

**FIGURE 4:**
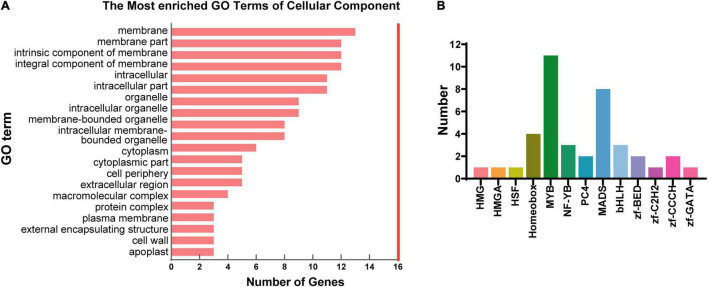
Analysis of differentially expressed genes and transcription factors (TFs) targeted by lncRNAs. **(A)** GO analysis of differentially expressed genes targeted by DElncRNAs. Top 20 terms of cellular component were displayed. **(B)** Number of TFs belonging to different families targeted by lncRNAs.

Among the target genes of DElncRNAs, 48 genes were identified to be differentially expressed (DEmRNAs) between N35 and N21. TopGO analysis of the DEmRNAs demonstrated that 13 of the 48 DEmRNAs were membrane associated (Figure 4A). Furthermore, six of the 13 DEmRNAs encoded membrane proteins related to transport function which are two casparian strip membrane proteins, the SNARE protein SYP132, an EamA domain protein, a nitrate transporter NRT1(PTR) and a transmembrane protein (Supplementary Table 4).

Since transcription factors (TFs) play important roles during nodule senescence, we investigated the TF genes targeted by DElncRNAs. Altogether, 41 TF genes belonging to 13 families were identified, of which, MYB (11, 26.8%) and MADS (8, 19.5%) constituted the largest two families containing the high number of target genes (Figure 4B). We found 7 of the 41 TF genes were differentially expressed between N21 and N35 including three MYB, one bHLH, one NF-YB, one TAZ and one AP2/ERF genes. Thus, we suggested that lncRNAs could be involved in nodule senescence mainly by targeting MYB transcription factors.

### Identification of Long Non-coding RNAs Targeting Well-Studied Genes Involved in Nodule Senescence

Since some ageing-related genes were reported to play important roles in nodule senescence (de Zelicourt et al., 2012; Berrabah et al., 2014; Pierre et al., 2014; Wang Q. et al., 2017; Dhanushkodi et al., 2018; Deng et al., 2019; Trujillo et al., 2019), the lncRNAs probably targeting them (within ± 100 kb of the associated genes) were investigated. We firstly examined the expression of these genes in the data of RNA-seq. As the early molecular markers of nodule senescence, two cysteine proteinase genes, *MtCP6* and *MtVPE*, were significantly up-regulated in N35. A NAC family TF gene *MtNAC969* which is a negative regulator of nodule senescence also showed up-regulated expression. While the expression of the rest genes has no significant difference between N21 and N35. The result was consistent with previous reports. A total of 34 lncRNAs were predicted targeting to 13 senescence-associated genes (Table 1). Of these, 9 DElncRNAs including 7 down-regulated and 2 up-regulated lncRNAs were identified. As seen from Table 1, most genes could be targeted by multiple lncRNAs. For instance, MtNAC*969* was probably targeted by six lncRNAs, two of them showed differential expression. *MtCP6* and *MtVPE* were targeted by one DElncRNAs, respectively. The result implied that lncRNAs played regulatory roles in nodule senescence by targeting some key senescence-related genes.

**TABLE 1:** The well-studied senescence-associated genes targeted by lncRNAs.

mRNA ID	Gene name	lncRNA ID	Senescence phenotype			References
			Mutant	Over expression	Knockout	
LOC25482000	VPE	MSTRG.393.1* MSTRG.404.1			RNAi Delay	Pierre et al. (2014)
LOC11406909	CP6	MSTRG.14829.1* MSTRG.14835.1 MSTRG.14835.2			RNAi Delay	Pierre et al. (2014)
LOC25492576	CP77	MSTRG.14233.1		Acceleration	CRISPR/Cas9 delay	Deng et al. (2019)
LOC11410578	bHLH2	MSTRG.18636.1 MSTRG.18640.9	T-DNA Early		TALEN Early	Deng et al. (2019)
LOC11443024	MtSymCRK	MSTRG.10509.1 MSTRG.10509.2* MSTRG.10509.3 MSTRG.10509.4 MSTRG.10512.6 MSTRG.10512.10 MSTRG.10514.1	Early			Berrabah et al. (2014)
LOC11445056	MtNAC969	MSTRG.14948.1* MSTRG.14948.6 MSTRG.14948.4* MSTRG.14948.5 MSTRG.14948.10 MSTRG.14948.11			RNAi Early	de Zelicourt et al. (2012)
newGene_5255	NFS2	MSTRG.28718.1 MSTRG.28719.1 MSTRG.28721.1 MSTRG.28721.3*		expression in A17 Early		Wang Q. et al. (2017)
LOC25487949	MtNPD2/4/5	MSTRG.7832.1 MSTRG.7833.2 MSTRG.7834.1* MSTRG.7847.2 MSTRG.7847.1* MSTRG.7847.5* MSTRG.7862.1			CRISPR/Cas9 Early	Trujillo et al. (2019)
LOC11432294	MtSer6	MSTRG.11553.2			RNAi Early	Dhanushkodi et al. (2018)
LOC11428040	MtFer2/Fer3	MSTRG.12828.1			RNAi Early	Dhanushkodi et al. (2018)

*Differentially expressed lncRNAs.

### Prediction of Differentially Expressed Long Non-coding RNAs Targeted by MicroRNAs

Plant lncRNAs can play regulatory roles by acting as the target mimicry of miRNA, so it is necessary to predict the senescence-associate lncRNAs targeting by miRNAs. In total, 49 DElncRNAs were predicted to be targeted by 93 miRNAs (Supplementary Table 5). We found that some miRNAs could target more than one DElncRNAs. As a well-known regulator of multiple physiological processes, miR156 probably target three DElncRNAs. In addition to miR156, some other miRNAs such as miR172 and miR168 were also found to interact with DElncRNAs. Conversely, some DElncRNAs could also bind multiple miRNAs. For example, MSTRG.16162.3 was predicted to bind with eight miRNAs which belonged to four miRNA families. High-throughput sequencing of miRNAs showed that 36 of the above 93 miRNAs were differentially expressed (Supplementary Table 5) including 19 known miRNAs such as miR156, miR172, miR1509 and miR2629, and 17 novel miRNAs.

### Most Long Non-coding RNAs Were Associated With Transposable Elements

**FIGURE 5:**
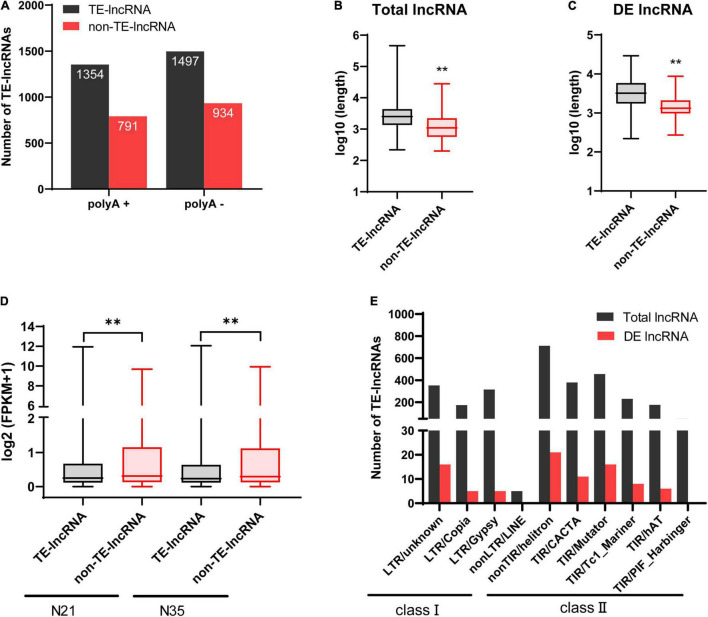
Characterizations of TE-lncRNAs. **(A)** The numbers of TE- and non-TE-lncRNAs in polyA+ and polyA-. **(B)** Average length of TE- and non-TE-lncRNAs in total lncRNAs. **(C)** Average length of TE- and non-TE-lncRNAs in DElncRNAs. **(D)** The expression levels (FPKM) of TE- and non-TE-lncRNAs. **(E)** Different TE families associated with total lncRNAs and DElncRNAs. ^**^p < 0.01(Wilcoxon test).

In plant, a large number of lncRNAs were originated from TEs and played important roles in plant development and abiotic stress responses. Therefore, lncRNAs associated with TEs (TE-lncRNAs) were identified. In total, there were 62.3% lncRNAs (2851) containing TEs including 87.7% lincRNAs (2499), 7% antisense (200), 3.9% intronic (112) and 1.4% sense lncRNAs (40; Table 2). For DE-lncRNAs, 87 (69%) lncRNAs were identified as TE-lncRNAs, of which lincRNAs accounted for 87.4%. Notably, 13 of the 17 DElncRNAs (76.5%) targeting memebrane-related genes were classified as TE-lncRNAs (Supplementary Table 4). The percentage of TE-lncRNAs in polyA+ RNA was closed to that in polyA-RNA (63.1% vs 61.6%; Figure 5A). Compared with non-TE-lncRNAs, the average length of TE-lncRNAs was significantly longer and their expression level was relatively lower (Figures 5B,D). Furthermore, for DElncRNAs, the difference in length between TE- and non-TE-lncRNAs was greater (Figure 5C). We investigated the family of TEs in lncRNAs according to their sequence homology with known TEs. In terms of quantity, the proportion of TE-lncRNAs associated with DNA transposons (2004, 70.3%) was much larger in *M. truncatula* than that in many plant species (Wang D. et al., 2017; Golicz et al., 2018b). The family which contributed the most to TE-lncRNAs was classified as Helitron, followed by TIR/Mutator, LTR/unknown, LTR/Gypsy and LTR/Copia. Similarly, for the TEs in DElncRNAs, the top three families were also Helitron, Mutator and LTR/unknown, but a small number of LTR/Copia and LTR/Gypsy elements were identified (Figure 5E).

**TABLE 2:** Summary of TE-lncRNAs.

	Number of TE-lncRNA	%TE-lncRNA	lincRNA	Intronic	Anti-sense	Sense
Total lncRNA	2851	62.3%	2499	112	200	40
DElncRNA	87	69%	76	2	8	1

In addition, we investigated the contribution of different TE families to lncRNA length (Table 3). We found that the lncRNAs related to LTR/Gypsy accounted for the most, which was different with the result calculated by quantity. Interestingly, the family that contributed most to DElncRNAs was TIR/Mutator rather than LTR/Gypsy. Furthermore, for both total lncRNAs and DElncRNAs, the contribution of DNA transposons to lncRNAs was greater than their contribution to the genome. Especially, DElncRNAs originated from TIR/Mutator accounted for 27.03% which was nearly three times the proportion of TIR/Mutator elements in all the TEs in the genome. However, the proportion of TE-lncRNAs derived from LTR/Gypsy or LTR/Copia was lower than their proportion in the genome.

**TABLE 3:** The contribution of different TE families to lncRNAs in quantity and length.

TE class	Total lncRNA		DE lncRNA		% total length of TE in genome
	Number of TE-lncRNAs	% total length of TE-lncRNAs	Number of TE-lncRNAs	% total length of TE-lncRNAs	
LTR/Copia	174	8.74	5	10.37	12.43
LTR/Gypsy	315	20.21	5	7.32	35
LTR/unknown	352	14.08	16	14.5	13.15
nonLTR/LINE	6	0.21	0	0	0.17
nonTIR/helitron	712	16.54	21	15.93	12.16
TIR/hAT	176	5.47	6	7.95	3.49
TIR/CACTA	379	11.12	11	10.5	10.22
TIR/Mutator	456	16.38	16	27.03	9.42
TIR/PIF_Harbinger	50	1.2	0	0	0.77
TIR/Tc1_Mariner	231	6.06	8	6.39	3.19

### Quantitative Real-Time Validation

**FIGURE 6:**
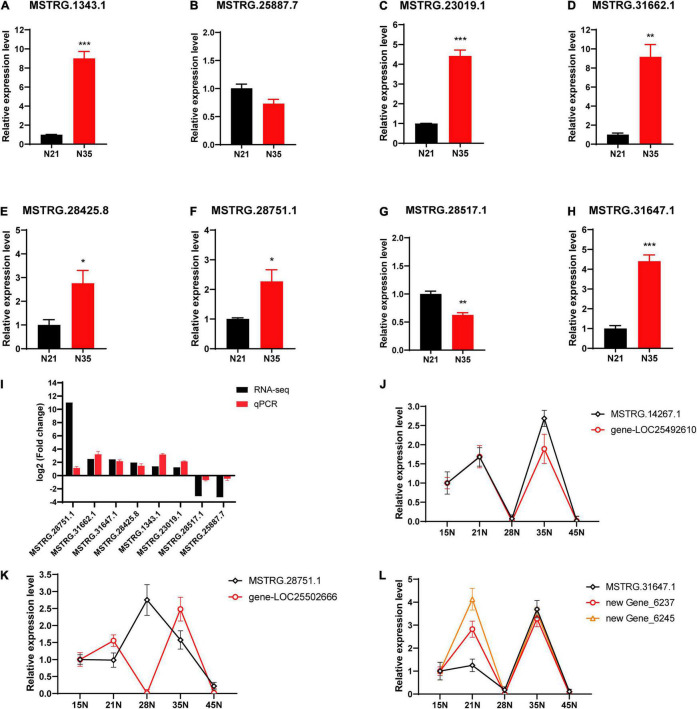
Expression validation of lncRNAs and their targets by qRT-PCR. **(A–H)** Validation of lncRNAs. *, ** and ***, respectively, represent difference significant at *p* < 0.05, *p* < 0.01 and *p* < 0.001. Error bars represent standard errors. **(I)** Comparison with the log2 (Fold change) for selected lncRNAs calculated by RNA-seq and qPCR. **(J–L)** Co-expression tendency of lncRNAs and their putative target genes. 15N, 21N, 28N, 35N, 45N represent nodules at 15, 21, 28, 35 and 45 dpi.

To verify the data of RNA-Seq, eight DElncRNAs were selected randomly for qRT-PCR detection (Figures 6A–H). The expression trends of the DElncRNAs were consistent with the results of RNA-Seq (Figure 6I), which indicated the reliability of expression analysis. Furthermore, we selected three interesting lncRNAs to check the co-expression tendency of lncRNAs and their putative target genes by qRT-PCR. In 15, 21, 28, 35, and 45 dpi nodules, the expression tendency of lncRNA MSTRG.14267.1 and gene-LOC25492610 (encoding a lysine-specific demethylase) was highly consistent (Figure 6J). While the expression profiles of lncRNA MSTRG28751.1 and gene-LOC25502666 (encoding a bHLH transcription factor) presented an opposite trend (Figure 6K). Additionally, the expression trends of two genes (senescence-associated gene, newGene_6237 and transmembrane protein gene, newGene_6245) were both consistent with that of MSTRG.31647.1 (Figure 6L).

## Discussion

### Long Non-coding RNAs Are Involved in Regulating Nodule Senescence

Nodule senescence leads to the decrease of nitrogen fixation efficiency and affect crop yield. Thus, one effective measure to ensure crop yield is to prolong the period of nitrogen fixation by delaying the onset of nodule senescence. Investigating the regulatory mechanism underlying nodule senescence can provide potential targets for such. However, little research has focused on the lncRNAs related to nodule senescence. In this study, we identified 126 putative nodule senescence-related lncRNAs by strand-specific RNA-seq. Our findings can provide insight into the functions of lncRNAs in nodule senescence and provided candidate targets for nodule senescence regulation.

### Transposable Element-Associated Long Non-coding RNAs Play Important Roles in Nodule Senescence

TEs are widely distributed in plant genome. Previous work has demonstrated that a number of plant lncRNAs are derived from TEs. Lots of TE-lncRNAs have been identified in Arabidopsis, rice (Wang D. et al., 2017), maize (Lv et al., 2019), cotton (Zhao et al., 2018), tomato (Wang et al., 2016) and soybean (Golicz et al., 2018b). Similarly, our results revealed that more than 60% lncRNAs contained TE sequences, and the proportion was higher in DElncRNAs. In many plant species, TE-lncRNAs mainly originate from retrotransposon especially LTR family (Wang D. et al., 2017). But surprisingly, we found in *M. truncatula* nodule, Helitron and TIR/Mutator families contributed the most to lncRNAs in quantity and length, respectively, which was distinct with the results in maize (Lv et al., 2019), rice (Wang D. et al., 2017), cotton (Zhao et al., 2018) and soybean (Golicz et al., 2018b), but have some similarities with the finding in Arabidopsis (Wang D. et al., 2017). The result suggested that the contribution of different TE families varied according to plant species and growth conditions. TEs act as the functional domain of lncRNAs (Johnson and Guigo, 2014) and current researches showed that TE-lncRNAs often work as regulators both in plant response to abiotic stress (Wang D. et al., 2017; Lv et al., 2019) and plant development including fruit ripening (Wang et al., 2016) and the control of seedling height (Zhao et al., 2018). However, whether they are involved in nodule senescence remained unknown. An interesting finding in this work is that the contribution of TIR/Mutator to DElncRNAs in length was significantly greater than that of other families, while Helitron contributed the most in quantity. Mutator elements were firstly found in maize and their homologues are distributed in many other plant species, which are called Mutator-like element (MULE). MULEs are able to selectively capture host gene fragments in Arabidopsis (Yu et al., 2000), maize (Talbert and Chandler, 1988) and rice (Juretic et al., 2005). Genes associated with MULEs play important roles in plant growth and development. For instance, in Arabidopsis MULE-derived genes acted as the transcriptional regulator of the genes involved in light response (Hudson et al., 2003). The mutation of genes related to MULEs caused delays in plant growth and flowering and reproductive defects (Joly-Lopez et al., 2012). Helitron elements have been reported to change the function and the expression level of genes (Hu et al., 2019; Liu et al., 2020). However, the contribution of MULE and Helitron to lncRNAs and plant aging is unknown. Our results suggested TE-lncRNAs derived from MULE and Helitron are involved in nodule senescence.

### Long Non-coding RNAs Regulate Nodule Senescence by Interacting With miRNAs

In plants, miRNAs are regarded as the control center of diverse biological processes including plant aging (Werner et al., 2021). During plant flowering, the aging pathway was regulated by miR156 and its target, SQUAMOSA PROMOTER BINDING-LIKE (SPL) transcription factors (Gou et al., 2019). SPL genes can increase the expression of miR172, and miR156/SPLs/miR172 constitute the regulatory network of aging pathway (Wei et al., 2017; Werner et al., 2021). Additionally, miR168 was involved in seed senescence in barley (Puchta et al., 2021). In legumes, miR156 and miR172 were essential regulators of nodulation in soybean (Yan et al., 2013; Yun et al., 2022), common bean (Nova-Franco et al., 2015) and alfalfa (Aung et al., 2017). However, the involvement of miRNAs in nodule senescence has not been reported. In our study, miR156, miR172 and miR168 displayed differential expression in N21 and N35, suggesting their roles in nodule senescence. LncRNAs can bind to miRNAs as competing endogenous targets. Here four DElncRNAs were predicted to be targeted by miR156 or miR172, which indicated that DElncRNAs could function in nodule aging by interacting with miRNAs.

### Long Non-coding RNAs Regulate Nodule Senescence by Affecting the Material Transport Across Membrane

TopGO analysis of DEmRNAs targeted by DElncRNAs showed that 13 DEmRNAs were associated with membrane component and six of them encoded proteins related to material transport. Of these proteins, SYP132, a Qa-SNARE, was reported to mediate the fusion between vesicles and the target cell membrane. A SNARE in tobacco was responsible for the regulation of cell membrane ion channels (Leyman et al., 1999). SYP132 in *A.thaliana* mediated the endocytosis of H^+^ATPase (Xia et al., 2019). Previous work suggested that MtSYP132 localized to the symbiosome membrane and the membrane around the infection threads, indicating its roles in nodulation and nodule development. The symbiosome membrane provides a medium for communication between the bacteroids and host cells and transmembrane ion transport across the symbiotic membrane is crucial for the function and survival of bacteroids (Catalano et al., 2007). Because MtSYP132 was up-regulated during senescence, it was likely to regulate the transport of some special aging-related molecules across the symbiosome membrane. Besides SYP132, two casparian strip membrane proteins and a NRT1(PTR) protein were also up-regulated. Casparian strip membrane proteins mediated the deposition of casparian strip which regulated the transport of water and inorganic salts, and defects in its development led to increased solute leakage (Wang et al., 2019; Calvo-Polanco et al., 2021). NRT1(PTR) proteins are known as nitrate transporter (Miller et al., 2007), which can also transport other substrates (Waterworth and Bray, 2006; Krouk et al., 2010). A NRT1(PTR) protein in *M. truncatula* was reported to be essential for lateral root growth and nodule development (Yendrek et al., 2010). In summary, we speculated that lncRNAs played a role in nodule senescence by affecting the material transport across membrane.

## Data Availability Statement

The data presented in the study are deposited in the SRA: https://www.ncbi.nlm.nih.gov/Traces/study/?acc=PRJNA810777, accession number PRJNA810777.

## Author Contributions

YL, LZ, and LY conceived the project and design the protocol. XQ, JY, and LY performed the experiments. LY, TH, TW, ZL, and YL performed the data analysis. YL and LZ wrote the manuscript. All authors contributed to the article and approved the submitted version.

## Conflict of Interest

The authors declare that the research was conducted in the absence of any commercial or financial relationships that could be construed as a potential conflict of interest.

## Publisher’s Note

All claims expressed in this article are solely those of the authors and do not necessarily represent those of their affiliated organizations, or those of the publisher, the editors and the reviewers. Any product that may be evaluated in this article, or claim that may be made by its manufacturer, is not guaranteed or endorsed by the publisher.

## Footnotes

1
https://github.com/oushujun/EDTA

2
ftp://ftp.ncbi.nih.gov/blast/db/

3
http://pfam.xfam.org/

4
http://www.ncbi.nlm.nih.gov/COG/

5
http://www.uniprot.org/

6
http://www.genome.jp/kegg/

7
http://www.geneontology.org/

## Supplementary Material

Supplementary Table 1 Primers for qPCR.

Supplementary Table 2 The statistics result of data quality control.

Supplementary Table 3 The mapping results of all samples.

Supplementary Table 4 Target genes of DElncRNAs enriched in membrane component.

Supplementary Table 5 DElncRNAs targeted by miRNAs.

Supplementary Figure 1 GO and KEGG analysis of target genes of DElncRNAs. **(A,B)** GO analysis of *cis* target genes of DElncRNAs. Top 20 terms of biological process **(A)** and molecular function **(B)** were displayed. **(C,D)** GO analysis of *trans* target genes of DElncRNAs. Top 20 terms of biological process **(C)** and molecular function **(D)** were displayed. **(E)** KEGG analysis of *cis* target genes of DElncRNAs. Top 20 pathways were displayed **(F)** KEGG analysis of *trans* target genes of DElncRNAs. Top 20 pathways were displayed.

## References

[B1] Anders S. Huber W. (2010). Differential expression analysis for sequence count data. *Genome Biol.* 11:R106. 10.1186/gb-2010-11-10-r106 20979621 PMC3218662

[B2] Aung B. Gao R. Gruber M. Y. Yuan Z. C. Sumarah M. Hannoufa A. (2017). MsmiR156 affects global gene expression and promotes root regenerative capacity and nitrogen fixation activity in alfalfa. *Transgenic Res.* 26 541–557. 10.1007/s11248-017-0024-3 28547343

[B3] Bardou F. Merchan F. Ariel F. Crespi M. (2011). Dual RNAs in plants. *Biochimie* 93 1950–1954. 10.1016/j.biochi.2011.07.028 21824505

[B4] Beattie J. M. (2014). Death by oxymoron? The enigma of heart failure with preserved ejection fraction. *Curr. Opin. Support Palliat. Care* 8 1–3. 10.1097/SPC.0000000000000035 24434724

[B5] Berrabah F. Bourcy M. Eschstruth A. Cayrel A. Guefrachi I. Mergaert P. (2014). A nonRD receptor-like kinase prevents nodule early senescence and defense-like reactions during symbiosis. *New Phytol.* 203 1305–1314. 10.1111/nph.12881 24916161

[B6] Calvo-Polanco M. Ribeyre Z. Dauzat M. Reyt G. Hidalgo-Shrestha C. Diehl P. (2021). Physiological roles of *Casparian strips* and suberin in the transport of water and solutes. *New Phytol.* 232 2295–2307. 10.1111/nph.17765 34617285 PMC9298204

[B7] Campalans A. Kondorosi A. Crespi M. (2004). Enod40, a short open reading frame-containing mRNA, induces cytoplasmic localization of a nuclear RNA binding protein in *Medicago truncatula*. *Plant Cell* 16 1047–1059. 10.1105/tpc.019406 15037734 PMC412876

[B8] Catalano C. M. Czymmek K. J. Gann J. G. Sherrier D. J. (2007). *Medicago truncatula* syntaxin SYP132 defines the symbiosome membrane and infection droplet membrane in root nodules. *Planta* 225 541–550. 10.1007/s00425-006-0369-y 16944200

[B9] Chao Y. Yuan J. Guo T. Xu L. Mu Z. Han L. (2019). Analysis of transcripts and splice isoforms in *Medicago sativa* L. by single-molecule long-read sequencing. *Plant Mol. Biol.* 99 219–235. 30600412 10.1007/s11103-018-0813-y

[B10] Chen L. Shi S. Jiang N. Khanzada H. Wassan G. M. Zhu C. (2018). Genome-wide analysis of long non-coding RNAs affecting roots development at an early stage in the rice response to cadmium stress. *BMC Genomics* 19:460. 29902991 10.1186/s12864-018-4807-6 PMC6002989

[B11] Csorba T. Questa J. I. Sun Q. Dean C. (2014). Antisense COOLAIR mediates the coordinated switching of chromatin states at FLC during vernalization. *Proc. Natl. Acad. Sci. U.S.A.* 111 16160–16165. 10.1073/pnas.1419030111 25349421 PMC4234544

[B12] Cui J. Jiang N. Hou X. Wu S. Zhang Q. Meng J. (2020). Genome-wide identification of lncRNAs and analysis of ceRNA networks during tomato resistance to *Phytophthora infestans*. *Phytopathology* 110 456–464. 10.1094/PHYTO-04-19-0137-R 31448997

[B13] de Zelicourt A. Diet A. Marion J. Laffont C. Ariel F. Moison M. (2012). Dual involvement of a *Medicago truncatula* NAC transcription factor in root abiotic stress response and symbiotic nodule senescence. *Plant J* 70 220–230. 10.1111/j.1365-313X.2011.04859.x 22098255

[B14] Deng J. Zhu F. Liu J. Zhao Y. Wen J. Wang T. (2019). Transcription factor bHLH2 represses cysteine protease77 to negatively regulate nodule senescence. *Plant Physiol.* 181 1683–1703. 10.1104/pp.19.00574 31591150 PMC6878008

[B15] Dhanushkodi R. Matthew C. McManus M. T. Dijkwel P. P. (2018). Drought-induced senescence of *Medicago truncatula* nodules involves serpin and ferritin to control proteolytic activity and iron levels. *New Phytol.* 220 196–208. 10.1111/nph.15298 29974467

[B16] Fahlgren N. Carrington J. C. (2010). miRNA target prediction in plants. *Methods Mol. Biol.* 592 51–57. 10.1007/978-1-60327-005-2_4 19802588

[B17] Fahraeus G. (1957). The infection of clover root hairs by nodule bacteria studied by a simple glass slide technique. *J. Gen. Microbiol.* 16 374–381. 10.1099/00221287-16-2-374 13416514

[B18] Golicz A. A. Singh M. B. Bhalla P. L. (2018b). The long intergenic noncoding RNA (LincRNA) landscape of the soybean genome. *Plant Physiol.* 176 2133–2147. 10.1104/pp.17.01657 29284742 PMC5841726

[B19] Golicz A. A. Bhalla P. L. Singh M. B. (2018a). lncRNAs in plant and animal sexual reproduction. *Trends Plant Sci.* 23 195–205. 10.1016/j.tplants.2017.12.009 29395831

[B20] Gou J. Tang C. Chen N. Wang H. Debnath S. Sun L. (2019). SPL7 and SPL8 represent a novel flowering regulation mechanism in switchgrass. *New Phytol.* 222 1610–1623. 10.1111/nph.15712 30688366

[B21] Hobecker K. V. Reynoso M. A. Bustos-Sanmamed P. Wen J. Mysore K. S. Crespi M. (2017). The MicroRNA390/TAS3 pathway mediates symbiotic nodulation and lateral root growth. *Plant Physiol.* 174 2469–2486. 10.1104/pp.17.00464 28663332 PMC5543954

[B22] Hu K. Xu K. Wen J. Yi B. Shen J. Ma C. (2019). Helitron distribution in *Brassicaceae* and whole Genome Helitron density as a character for distinguishing plant species. *BMC Bioinformatics* 20:354. 10.1186/s12859-019-2945-8 31234777 PMC6591975

[B23] Huang X. Zhang H. Wang Q. Guo R. Wei L. Song H. (2021). Genome-wide identification and characterization of long non-coding RNAs involved in flag leaf senescence of rice. *Plant Mol. Biol.* 105 655–684. 10.1007/s11103-021-01121-3 33569692 PMC7985109

[B24] Hudson M. E. Lisch D. R. Quail P. H. (2003). The FHY3 and FAR1 genes encode transposase-related proteins involved in regulation of gene expression by the phytochrome A-signaling pathway. *Plant J.* 34 453–471. 10.1046/j.1365-313x.2003.01741.x 12753585

[B25] Johnson R. Guigo R. (2014). The RIDL hypothesis: transposable elements as functional domains of long noncoding RNAs. *RNA* 20 959–976. 10.1261/rna.044560.114 24850885 PMC4114693

[B26] Joly-Lopez Z. Forczek E. Hoen D. R. Juretic N. Bureau T. E. (2012). A gene family derived from transposable elements during early angiosperm evolution has reproductive fitness benefits in *Arabidopsis thaliana*. *PLoS Genet.* 8:e1002931. 10.1371/journal.pgen.1002931 22969437 PMC3435246

[B27] Juretic N. Hoen D. R. Huynh M. L. Harrison P. M. Bureau T. E. (2005). The evolutionary fate of MULE-mediated duplications of host gene fragments in rice. *Genome Res.* 15 1292–1297. 10.1101/gr.4064205 16140995 PMC1199544

[B28] Kim D. Paggi J. M. Park C. Bennett C. Salzberg S. L. (2019). Graph-based genome alignment and genotyping with HISAT2 and HISAT-genotype. *Nat. Biotechnol.* 37 907–915. 10.1038/s41587-019-0201-4 31375807 PMC7605509

[B29] Kong L. Zhang Y. Ye Z. Q. Liu X. Q. Zhao S. Q. Wei L. (2007). CPC: assess the protein-coding potential of transcripts using sequence features and support vector machine. *Nucleic Acids Res.* 35 W345–W349. 10.1093/nar/gkm391 17631615 PMC1933232

[B30] Krouk G. Lacombe B. Bielach A. Perrine-Walker F. Malinska K. Mounier E. (2010). Nitrate-regulated auxin transport by NRT1.1 defines a mechanism for nutrient sensing in plants. *Dev. Cell* 18 927–937. 10.1016/j.devcel.2010.05.008 20627075

[B31] Leyman B. Geelen D. Quintero F. J. Blatt M. R. (1999). A tobacco syntaxin with a role in hormonal control of guard cell ion channels. *Science* 283 537–540. 10.1126/science.283.5401.537 9915701

[B32] Li L. Eichten S. R. Shimizu R. Petsch K. Yeh C. T. Wu W. (2014). Genome-wide discovery and characterization of maize long non-coding RNAs. *Genome Biol.* 15:R40. 10.1186/gb-2014-15-2-r40 24576388 PMC4053991

[B33] Liu J. Jung C. Xu J. Wang H. Deng S. Bernad L. (2012). Genome-wide analysis uncovers regulation of long intergenic noncoding RNAs in *Arabidopsis*. *Plant Cell* 24 4333–4345. 10.1105/tpc.112.102855 23136377 PMC3531837

[B34] Liu Q. Deng S. Liu B. Tao Y. Ai H. Liu J. (2020). A helitron-induced RabGDIalpha variant causes quantitative recessive resistance to maize rough dwarf disease. *Nat. Commun.* 11:495. 10.1038/s41467-020-14372-3 31980630 PMC6981192

[B35] Lv Y. Hu F. Zhou Y. Wu F. Gaut B. S. (2019). Maize transposable elements contribute to long non-coding RNAs that are regulatory hubs for abiotic stress response. *BMC Genomics* 20:864. 10.1186/s12864-019-6245-5 31729949 PMC6858665

[B36] Ma X. Zhang X. Traore S. M. Xin Z. Ning L. Li K. (2020). Genome-wide identification and analysis of long noncoding RNAs (lncRNAs) during seed development in peanut (*Arachis hypogaea* L.). *BMC Plant Biol.* 20:192. 10.1186/s12870-020-02405-4 32375650 PMC7203998

[B37] Mao X. Cai T. Olyarchuk J. G. Wei L. (2005). Automated genome annotation and pathway identification using the KEGG Orthology (KO) as a controlled vocabulary. *Bioinformatics* 21 3787–3793. 10.1093/bioinformatics/bti430 15817693

[B38] Miller A. J. Fan X. Orsel M. Smith S. J. Wells D. M. (2007). Nitrate transport and signalling. *J. Exp. Bot.* 58 2297–2306. 10.1093/jxb/erm066 17519352

[B39] Nova-Franco B. Iniguez L. P. Valdes-Lopez O. Alvarado-Affantranger X. Leija A. Fuentes S. I. (2015). The micro-RNA72c-APETALA2-1 node as a key regulator of the common bean-Rhizobium etli nitrogen fixation symbiosis. *Plant Physiol.* 168 273–291. 10.1104/pp.114.255547 25739700 PMC4424015

[B40] Ou S. Su W. Liao Y. Chougule K. Agda J. R. A. Hellinga A. J. (2019). Benchmarking transposable element annotation methods for creation of a streamlined, comprehensive pipeline. *Genome Biol.* 20:275. 10.1186/s13059-019-1905-y 31843001 PMC6913007

[B41] Pecrix Y. Staton S. E. Sallet E. Lelandais-Briere C. Moreau S. Carrere S. (2018). Whole-genome landscape of *Medicago truncatula* symbiotic genes. *Nat. Plants* 4 1017–1025. 10.1038/s41477-018-0286-7 30397259

[B42] Pertea M. Pertea G. M. Antonescu C. M. Chang T. C. Mendell J. T. Salzberg S. L. (2015). StringTie enables improved reconstruction of a transcriptome from RNA-seq reads. *Nat. Biotechnol.* 33 290–295. 10.1038/nbt.3122 25690850 PMC4643835

[B43] Pierre O. Hopkins J. Combier M. Baldacci F. Engler G. Brouquisse R. (2014). Involvement of papain and legumain proteinase in the senescence process of *Medicago truncatula* nodules. *New Phytol.* 202 849–863. 10.1111/nph.12717 24527680

[B44] Puchta M. Groszyk J. Malecka M. Koter M. D. Niedzielski M. Rakoczy-Trojanowska M. (2021). Barley seeds miRNome stability during long-term storage and aging. *Int. J. Mol. Sci.* 22:4315. 10.3390/ijms22094315 33919202 PMC8122619

[B45] Rai M. I. Alam M. Lightfoot D. A. Gurha P. Afzal A. J. (2019). Classification and experimental identification of plant long non-coding RNAs. *Genomics* 111 997–1005. 10.1016/j.ygeno.2018.04.014 29679643

[B46] Sun L. Luo H. Bu D. Zhao G. Yu K. Zhang C. (2013). Utilizing sequence intrinsic composition to classify protein-coding and long non-coding transcripts. *Nucleic Acids Res.* 41:e166. 10.1093/nar/gkt646 23892401 PMC3783192

[B47] Talbert L. E. Chandler V. L. (1988). Characterization of a highly conserved sequence related to mutator transposable elements in maize. *Mol. Biol. Evol.* 5 519–529. 10.1093/oxfordjournals.molbev.a040510 2848175

[B48] Trapnell C. Williams B. A. Pertea G. Mortazavi A. Kwan G. van Baren M. J. (2010). Transcript assembly and quantification by RNA-Seq reveals unannotated transcripts and isoform switching during cell differentiation. *Nat. Biotechnol.* 28 511–515. 10.1038/nbt.1621 20436464 PMC3146043

[B49] Trujillo D. I. Silverstein K. A. T. Young N. D. (2019). Nodule-specific PLAT domain proteins are expanded in the *Medicago lineage* and required for nodulation. *New Phytol.* 222 1538–1550. 10.1111/nph.15697 30664233

[B50] Van de Velde W. Guerra J. C. De Keyser A. De Rycke R. Rombauts S. Maunoury N. (2006). Aging in legume symbiosis. A molecular view on nodule senescence in *Medicago truncatula*. *Plant Physiol.* 141 711–720. 10.1104/pp.106.078691 16648219 PMC1475454

[B51] Wang D. Qu Z. Yang L. Zhang Q. Liu Z. H. Do T. (2017). Transposable elements (TEs) contribute to stress-related long intergenic noncoding RNAs in plants. *Plant J.* 90 133–146. 10.1111/tpj.13481 28106309 PMC5514416

[B52] Wang H. Niu Q. W. Wu H. W. Liu J. Ye J. Yu N. (2015). Analysis of non-coding transcriptome in rice and maize uncovers roles of conserved lncRNAs associated with agriculture traits. *Plant J.* 84 404–416. 10.1111/tpj.13018 26387578

[B53] Wang L. Park H. J. Dasari S. Wang S. Kocher J. P. Li W. (2013). CPAT: coding-potential assessment tool using an alignment-free logistic regression model. *Nucleic Acids Res.* 41:e74. 10.1093/nar/gkt006 23335781 PMC3616698

[B54] Wang M. Yuan D. Tu L. Gao W. He Y. Hu H. (2015). Long noncoding RNAs and their proposed functions in fibre development of cotton (*Gossypium* spp.). *New Phytol.* 207 1181–1197. 10.1111/nph.13429 25919642

[B55] Wang Q. Yang S. Liu J. Terecskei K. Abraham E. Gombar A. (2017). Host-secreted antimicrobial peptide enforces symbiotic selectivity in *Medicago truncatula*. *Proc. Natl. Acad. Sci. U.S.A.* 114 6854–6859. 10.1073/pnas.1700715114 28607058 PMC5495241

[B56] Wang T. Z. Zhao M. G. Zhang X. X. Liu M. Yang C. G. Chen Y. H. (2017). Novel phosphate deficiency-responsive long non-coding RNAs in the legume model plant *Medicago truncatula*. *J. Exp. Bot.* 68 5937–5948. 10.1093/jxb/erx384 29165588 PMC5854128

[B57] Wang X. Ai G. Zhang C. Cui L. Wang J. Li H. (2016). Expression and diversification analysis reveals transposable elements play important roles in the origin of *Lycopersicon*-specific lncRNAs in tomato. *New Phytol.* 209 1442–1455. 10.1111/nph.13718 26494192

[B58] Wang Y. Q. Fan X. D. Lin F. He G. M. Terzaghi W. Zhu D. M. (2014). Arabidopsis noncoding RNA mediates control of photomorphogenesis by red light. *Proc. Natl. Acad. Sci. U.S.A.* 111 10359–10364. 10.1073/pnas.1409457111 24982146 PMC4104870

[B59] Wang Z. Yamaji N. Huang S. Zhang X. Shi M. Fu S. (2019). OsCASP1 is required for casparian strip formation at endodermal cells of rice roots for selective uptake of mineral elements. *Plant Cell* 31 2636–2648. 10.1105/tpc.19.00296 31484684 PMC6881135

[B60] Waterworth W. M. Bray C. M. (2006). Enigma variations for peptides and their transporters in higher plants. *Ann. Bot.* 98 1–8. 10.1093/aob/mcl099 16735405 PMC2803549

[B61] Wei Q. Ma C. Xu Y. Wang T. Chen Y. Lu J. (2017). Control of chrysanthemum flowering through integration with an aging pathway. *Nat. Commun.* 8:829. 10.1038/s41467-017-00812-0 29018260 PMC5635119

[B62] Werner S. Bartrina I. Schmulling T. (2021). Cytokinin regulates vegetative phase change in *Arabidopsis thaliana* through the miR172/TOE1-TOE2 module. *Nat. Commun.* 12:5816. 10.1038/s41467-021-26088-z 34611150 PMC8492644

[B63] Xia L. Mar Marques-Bueno M. Bruce C. G. Karnik R. (2019). Unusual roles of secretory SNARE SYP132 in plasma membrane H(+)-ATPase traffic and vegetative plant growth. *Plant Physiol.* 180 837–858. 10.1104/pp.19.00266 30926657 PMC6548232

[B64] Yan Z. Hossain M. S. Wang J. Valdes-Lopez O. Liang Y. Libault M. (2013). miR172 regulates soybean nodulation. *Mol. Plant Microbe Interact.* 26 1371–1377. 10.1094/MPMI-04-13-0111-R 23980625

[B65] Yendrek C. R. Lee Y. C. Morris V. Liang Y. Pislariu C. I. Burkart G. (2010). A putative transporter is essential for integrating nutrient and hormone signaling with lateral root growth and nodule development in *Medicago truncatula*. *Plant J.* 62 100–112. 10.1111/j.1365-313X.2010.04134.x 20088899

[B66] Yu Z. Wright S. I. Bureau T. E. (2000). Mutator-like elements in *Arabidopsis thaliana*. Structure, diversity and evolution. *Genetics* 156 2019–2031. 10.1093/genetics/156.4.2019 11102392 PMC1461377

[B67] Yun J. Sun Z. Jiang Q. Wang Y. Wang C. Luo Y. (2022). The miR156b-GmSPL9d module modulates nodulation by targeting multiple core nodulation genes in soybean. *New Phytol.* 233 1881–1899. 10.1111/nph.17899 34862970 PMC9303946

[B68] Zhao T. Tao X. Feng S. Wang L. Hong H. Ma W. (2018). LncRNAs in polyploid cotton interspecific hybrids are derived from transposon neofunctionalization. *Genome Biol.* 19:195. 10.1186/s13059-018-1574-2 30419941 PMC6233382

[B69] Zhu B. Yang Y. Li R. Fu D. Wen L. Luo Y. (2015). RNA sequencing and functional analysis implicate the regulatory role of long non-coding RNAs in tomato fruit ripening. *J. Exp. Bot.* 66 4483–4495. 10.1093/jxb/erv203 25948705 PMC4507755

