# Supplementary material for: Genome-Wide Analysis of Long Non-coding RNAs Involved in Nodule Senescence in Medicago truncatula
Source: Front Plant Sci. 2022 May 30;13:917840. doi: 10.3389/fpls.2022.917840 (PMC9189404; doi:10.3389/fpls.2022.917840)
Supplement: Supplementary Table 1 — Primers for qPCR. [file Table_1.docx]

**Supplementary Table 1 Primers for qPCR**

| LncRNA/gene ID | Primer |
| --- | --- |
| MSTRG.1343.1 | TTCGCACAATTGGTCATCGC |
|  | TTCTACCCGCCGCTCAATAG |
| MSTRG.13427.7 | ACCCTTCCACATGCTTGTAAC |
|  | AGCTTCGTGCTGATAACGTGT |
| MSTRG.25887.7 | ACGCCGTCTTGAAAGGACAT |
|  | AGAGGGGTATCTGTTCGCCT |
| MSTRG.23019.1 | CACCGTTGCCAAAAGGACAG |
|  | ACCATGATCAACATGACCCATCA |
| MSTRG.31662.1 | AATCCGGGCTAGAAGCGATG |
|  | GATTCTACCCGCCGCTCAAT |
| MSTRG.14267.1 | ACCCTTGAGCTTGAGGTTTCT |
|  | CGCTTTCCACGTTTCTCAGC |
| MSTRG.28425.8 | TGGATCGAGCACCAAGCATA |
|  | TGTTGTCTATTGTTAGTAGCGTGC |
| MSTRG.28517.1 | GAAGCTAGGAAGAGGGCTGC |
|  | TCCAGCTAGGGATGAACCGA |
| MSTRG.28751.1 | CTCCGTCCAGTTGCCTCTTC |
|  | CTGAGCCATCACTCAACGGT |
| MSTRG.31647.1 | AATCCGGGCTAGAAGCGATG |
|  | CAAGGCCACTCTGCCACTTA |
| LOC25492610 | GCTGCCAGCGCACCAT |
|  | GGTGCAGTGGAAAGCAAAGG |
| LOC25502666 | GGGGACCATTTTGGAGCACA |
|  | ATCCAACACTCACTCACTCTTCT |
| Medicago_truncatula_newGene_6237 | AAAATCTCCACGAGGCACGG |
|  | AGGGGAAGGTCTAGTCCAGG |
| Medicago_truncatula_newGene_6245 | AAACTGGAAATGCCTCGTCT |
| *Mtactin11* | CCTGCACTGCTGGTAAGAA  ACGAGCGTTTCAGATG  ACCTCCGATCCAGACA |

**Supplementary Table 2. The statistics result of data quality control**

| **Sample Name** | **Read Sum** | **Base Sum** | **Q20(%)** | **Q30(%)** | **GC Content (%)** |
| --- | --- | --- | --- | --- | --- |
| N21-1 | 55,596,472 | 16,464,121,434 | 97.54 | 92.82 | 44.14 |
| N21-2 | 71,305,005 | 21,132,919,520 | 97.31 | 92.4 | 44.78 |
| N21-3 | 64,383,315 | 19,048,531,882 | 97.25 | 92.26 | 44.47 |
| N35-1 | 56,482,469 | 16,736,278,044 | 98 | 94.68 | 43.23 |
| N35-2 | 59,338,818 | 17,629,659,928 | 98.58 | 95.69 | 44.75 |
| N35-3 | 57,786,700 | 17,102,171,292 | 97.95 | 94.65 | 44.47 |

**Supplementary Table 3 The mapping results of all samples**

| **Sample Name** | **Total Reads** | **Mapped Reads** | **Uniq Mapped Reads** | **Multiple Mapped Reads** |
| --- | --- | --- | --- | --- |
| N21-1 | 111,192,944 | 81,309,420  (73.12%) | 76,450,121  (68.75%) | 4,859,299  (4.37%) |
| N21-2 | 142,610,010 | 100,775,450  (70.67%) | 94,585,417  (66.32%) | 6,190,033  (4.34%) |
| N21-3 | 128,766,630 | 92,769,697  (72.04%) | 87,050,058  (67.60%) | 5,719,639  (4.44%) |
| N35-1 | 112,964,938 | 89,125,630  (78.90%) | 83,770,033  (74.16%) | 5,355,597  (4.74%) |
| N35-2 | 118,677,636 | 88,791,801  (74.82%) | 83,486,300  (70.35%) | 5,305,501  (4.47%) |
| N35-3 | 115,573,400 | 83,656,578  (72.38%) | 78,465,244  (67.89%) | 5,191,334  (4.49%) |

**Supplementary Table 4 Target genes of DElncRNAs enriched in membrane component**

| LncRNA ID | Gene ID | Annotation |
| --- | --- | --- |
| *MSTRG.16162.3* | gene-LOC11439135 | LRR receptor-like serine/threonine-protein kinase FEI 1 |
| *MSTRG.14948.1*  *MSTRG.14948.4* | gene-LOC11446913 | casparian strip membrane protein 2 |
| MSTRG.31647.1  MSTRG.31662.1  MSTRG.31667.1 | newGene_6245 | transmembrane protein |
| *MSTRG.12889.2* | gene-LOC11422473 | chlorophyll A-B binding protein |
| *MSTRG.22399.4* | newGene_3389 | Nodule Cysteine-Rich (NCR) secreted peptide |
| *MSTRG.21779.1* | gene-LOC25495863 | syntaxin-132 |
| *MSTRG.17961.1* | gene-LOC11408558 | cytochrome P450 81E8 |
| MSTRG.31620.1 | gene-LOC25483783 | casparian strip membrane protein 1 |
| *MSTRG.8451.10*  *MSTRG.8451.12* | gene-LOC11418309 | cytochrome P450 family protein |
| *MSTRG.18814.2* | newGene_2204 | putative EamA domain-containing protein |
| *MSTRG.18770.4* | gene-LOC11408630 | cysteine-rich receptor-like protein kinase 2 |
| *MSTRG.6569.6* | gene-LOC11446770 | photosystem II 10 kDa polypeptide |
| *MSTRG.9593.6* | gene-LOC25490793 | protein NRT1/ PTR FAMILY 8.1 |

Italics represent TE-lncRNAs.

**Supplementary Table 5 DElncRNAs targeted by miRNAs**

|  | | | | | | | |
| --- | --- | --- | --- | --- | --- | --- | --- |
| DElncRNA ID | micro RNA | | | | | | |
| MSTRG.28425.8 | mtr-miR156h-5p | mtr-miR156e | mtr-miR7701-5p | mtr-miR2619b-3p | *mtr-miR156g-5p* | mtr-miR156f |  |
| MSTRG.28425.6 | mtr-miR156h-5p | mtr-miR156e | mtr-miR7701-5p | mtr-miR2619b-3p | *mtr-miR156g-5p* | mtr-miR156f |  |
| MSTRG.28425.7 | mtr-miR156h-5p | mtr-miR156e | mtr-miR7701-5p | mtr-miR2619b-3p | *mtr-miR156g-5p* | mtr-miR156f |  |
| MSTRG.821.3 | miR2676a | mtr-miR2676f | mtr-miR2676e | mtr-miR2676d | *novel_miR131* | mtr-miR2676c | mtr-miR2676b |
| MSTRG.28424.7 | mtr-miR2588b | mtr-miR2588a |  |  |  |  |  |
| MSTRG.25887.7 | mtr-miR2593c | mtr-miR7701-5p | mtr-miR2593b | *mtr-miR5205a* | mtr-miR2593a |  |  |
| MSTRG.4205.2 | *novel_miR102* | novel_miR98 | *mtr-miR2596* | *novel_miR212* |  |  |  |
| MSTRG.20924.2 | *mtr-miR2629b* | mtr-miR2627 | mtr-miR2629f | *mtr-miR2629a* | *mtr-miR2629g* | *mtr-miR2629c* | *mtr-miR2629e* |
| MSTRG.14948.1 | mtr-miR5227 |  |  |  |  |  |  |
| MSTRG.14948.4 | mtr-miR5227 |  |  |  |  |  |  |
| MSTRG.7485.1 | *miR172a* | mtr-miR5237 | mtr-miR172c-5p | mtr-miR172d-3p | mtr-miR172c-3p | mtr-miR172b |  |
| MSTRG.6197.1 | mtr-miR2638a | mtr-miR2638b | novel_miR194 | novel_miR153 |  |  |  |
| MSTRG.12718.3 | mtr-miR2644 |  |  |  |  |  |  |
| MSTRG.12889.2 | mtr-miR5748 |  |  |  |  |  |  |
| MSTRG.3870.34 | *novel_miR64* |  |  |  |  |  |  |
| MSTRG.8451.10 | *novel_miR49* | novel_miR168 | novel_miR174 | novel_miR92 | *novel_miR50* | *novel_miR96* | *novel_miR86* |
| MSTRG.25981.6 | *mtr-miR5758* | mtr-miR5241a | mtr-miR5241c | mtr-miR5241b |  |  |  |
| MSTRG.24186.1 | mtr-miR7701-5p |  |  |  |  |  |  |
| MSTRG.18770.4 | mtr-miR7701-5p | *mtr-miR5559-5p* |  |  |  |  |  |
| MSTRG.32007.1 | mtr-miR7701-5p |  |  |  |  |  |  |
| MSTRG.8340.2 | mtr-miR5561-5p | *mtr-miR5561-3p* |  |  |  |  |  |
| MSTRG.8451.12 | novel_miR168 | novel_miR92 | novel_miR96 |  |  |  |  |
| MSTRG.27395.1 | *novel_miR24* | *mtr-miR1509a-5p* | *mtr-miR1509b* |  |  |  |  |
| MSTRG.28335.4 | *novel_miR24* | *mtr-miR2598* | mtr-miR2590c |  |  |  |  |
| MSTRG.29206.3 | *novel_miR24* | *mtr-miR5215* | *mtr-miR5260* | *mtr-miR1509b* |  |  |  |
| MSTRG.6569.6 | *mtr-miR2586a* |  |  |  |  |  |  |
| MSTRG.13580.17 | *novel_miR23* |  |  |  |  |  |  |
| DElncRNA ID | micro RNA | | | | | | |
| MSTRG.13580.1 | *novel_miR23* |  |  |  |  |  |  |
| MSTRG.23000.3 | mtr-miR5741a | novel_miR6 | mtr-miR5741e | mtr-miR5741b | mtr-miR5741d | *novel_miR138* | mtr-miR5741c |
| MSTRG.23000.4 | mtr-miR5741a | novel_miR6 | mtr-miR5741e | mtr-miR5741b | mtr-miR5741d | *novel_miR138* | mtr-miR5741c |
| MSTRG.23000.8 | mtr-miR5741a | mtr-miR5741e | mtr-miR5741b | mtr-miR5741d | mtr-miR5741c |  |  |
| MSTRG.16162.3 | mtr-miR5741a | mtr-miR5741e | mtr-miR5741b | mtr-miR2655b | mtr-miR5741d | mtr-miR7699-5p | mtr-miR5741c |
| MSTRG.17167.1 | novel_miR98 | mtr-miR5277 |  |  |  |  |  |
| MSTRG.31882.4 | *novel_miR26* | *novel_miR91* |  |  |  |  |  |
| MSTRG.29248.2 | *novel_miR26* | mtr-miR5298a |  |  |  |  |  |
| MSTRG.13723.4 | mtr-miR2645 | novel_miR152 | *novel_miR38* |  |  |  |  |
| MSTRG.4350.20 | *novel_miR152* | novel_miR199 | *novel_miR38* |  |  |  |  |
| MSTRG.18451.2 | *novel_miR149* |  |  |  |  |  |  |
| MSTRG.612.8 | *novel_miR30* |  |  |  |  |  |  |
| MSTRG.17921.3 | *novel_miR30* |  |  |  |  |  |  |
| MSTRG.6194.4 | mtr-miR2655b |  |  |  |  |  |  |
| MSTRG.12031.3 | mtr-miR2608 |  |  |  |  |  |  |
| MSTRG.18340.5 | mtr-miR2600a |  |  |  |  |  |  |
| MSTRG.28553.31 | mtr-miR5217 |  |  |  |  |  |  |
| MSTRG.28553.23 | mtr-miR5217 |  |  |  |  |  |  |
| MSTRG.28554.6 | mtr-miR5217 |  |  |  |  |  |  |
| MSTRG.30368.1 | *mtr-miR2634* |  |  |  |  |  |  |
| MSTRG.18511.3 | mtr-miR5240 |  |  |  |  |  |  |
| MSTRG.31147.1 | mtr-miR5240 |  |  |  |  |  |  |

Italics represent differentially expressed miRNAs identified by high-throughput sequencing.
