# Supplementary figures and images for: Genome-Wide Analysis of Long Non-coding RNAs Involved in Nodule Senescence in Medicago truncatula
Source: Front Plant Sci. 2022 May 30;13:917840. doi: 10.3389/fpls.2022.917840 (PMC9189404; doi:10.3389/fpls.2022.917840)

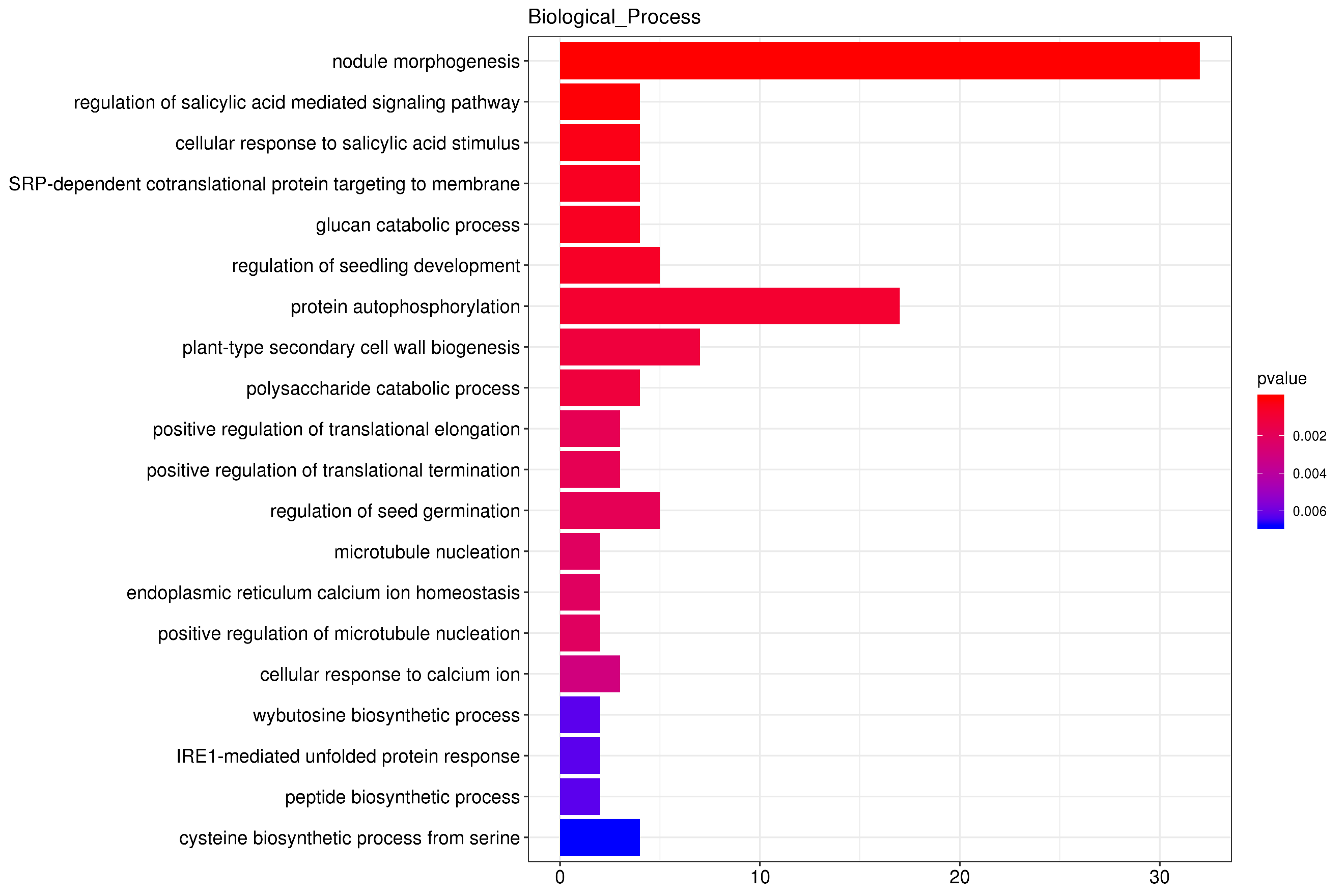


A

B


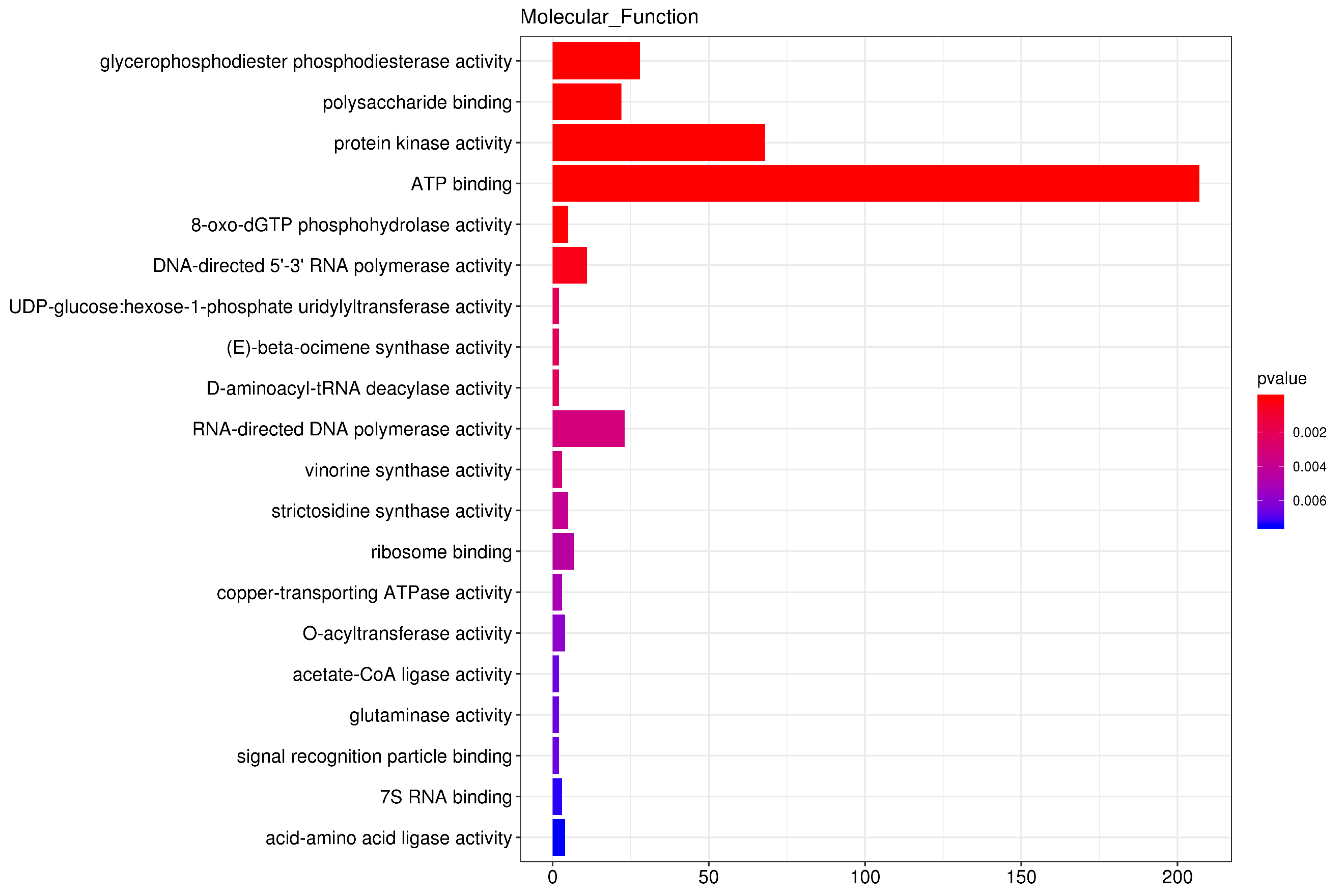


C


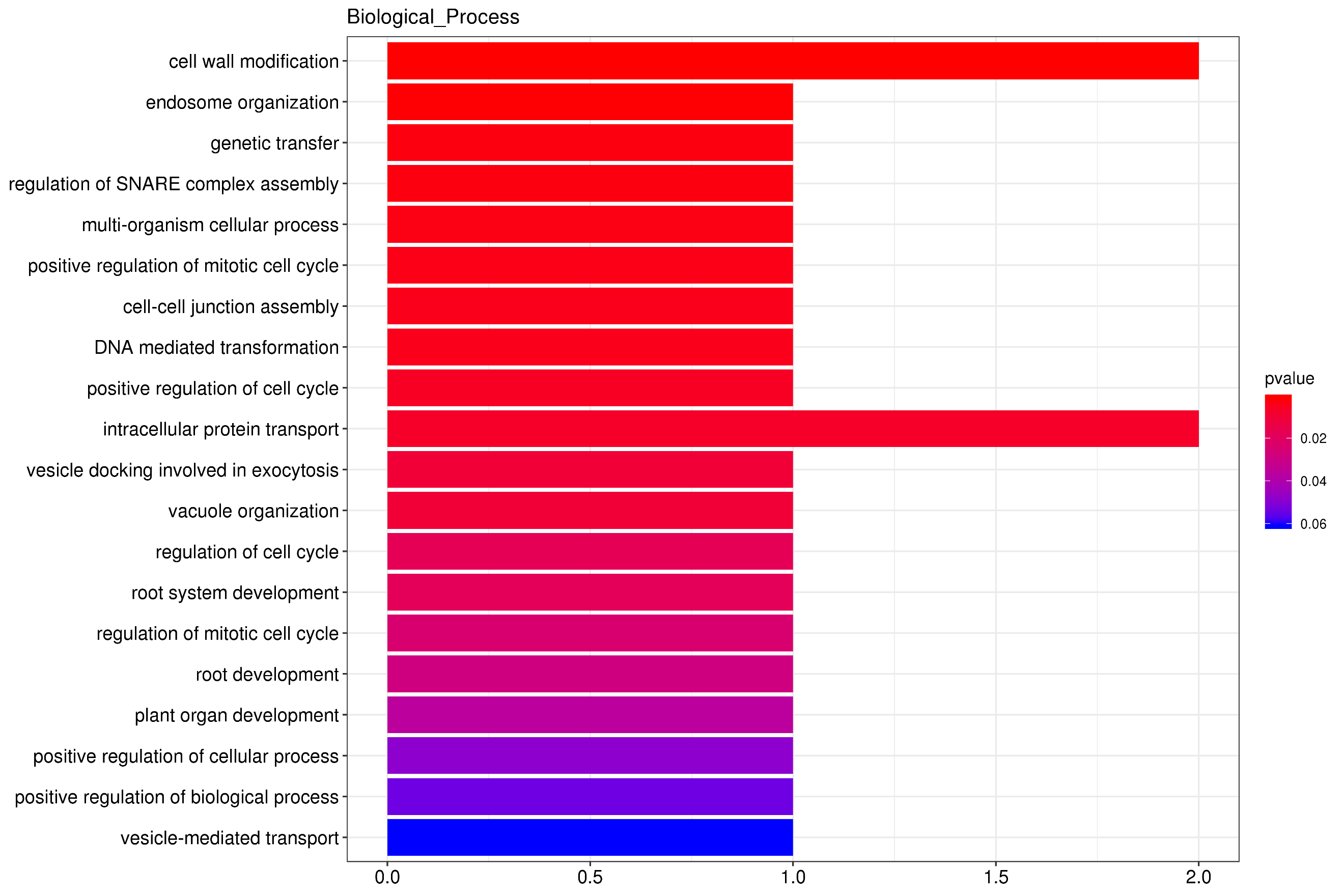


D

E

F

D


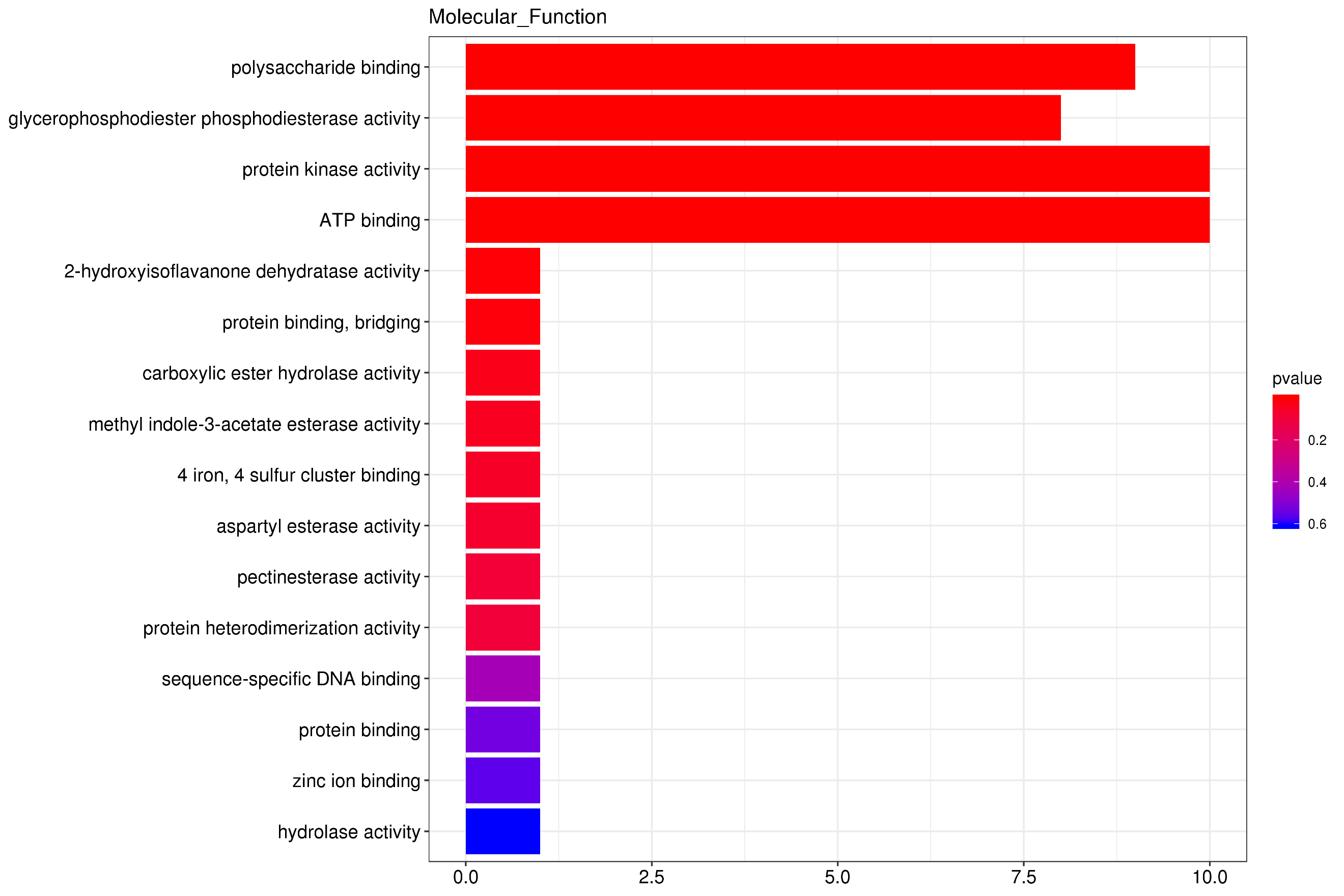


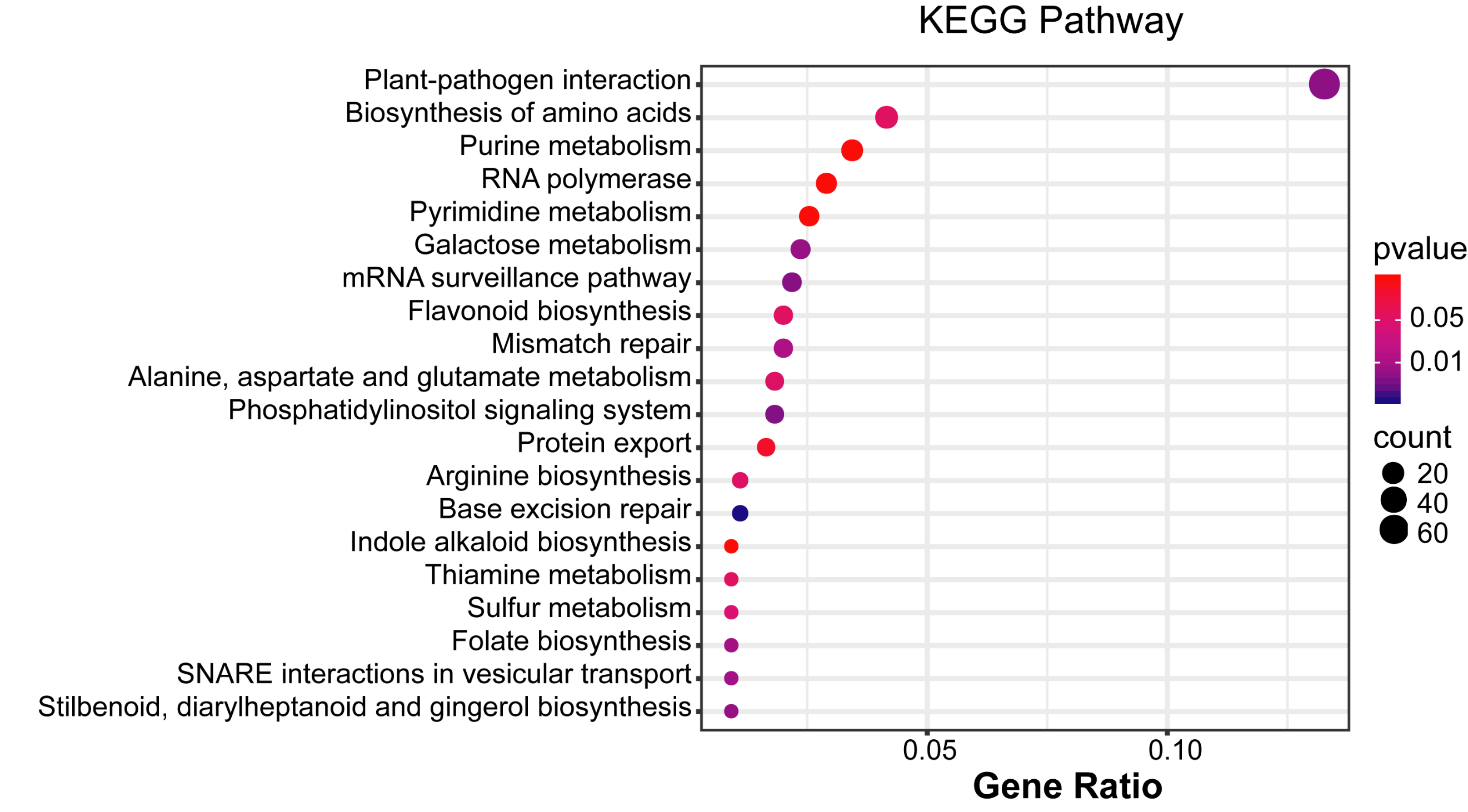


E

F


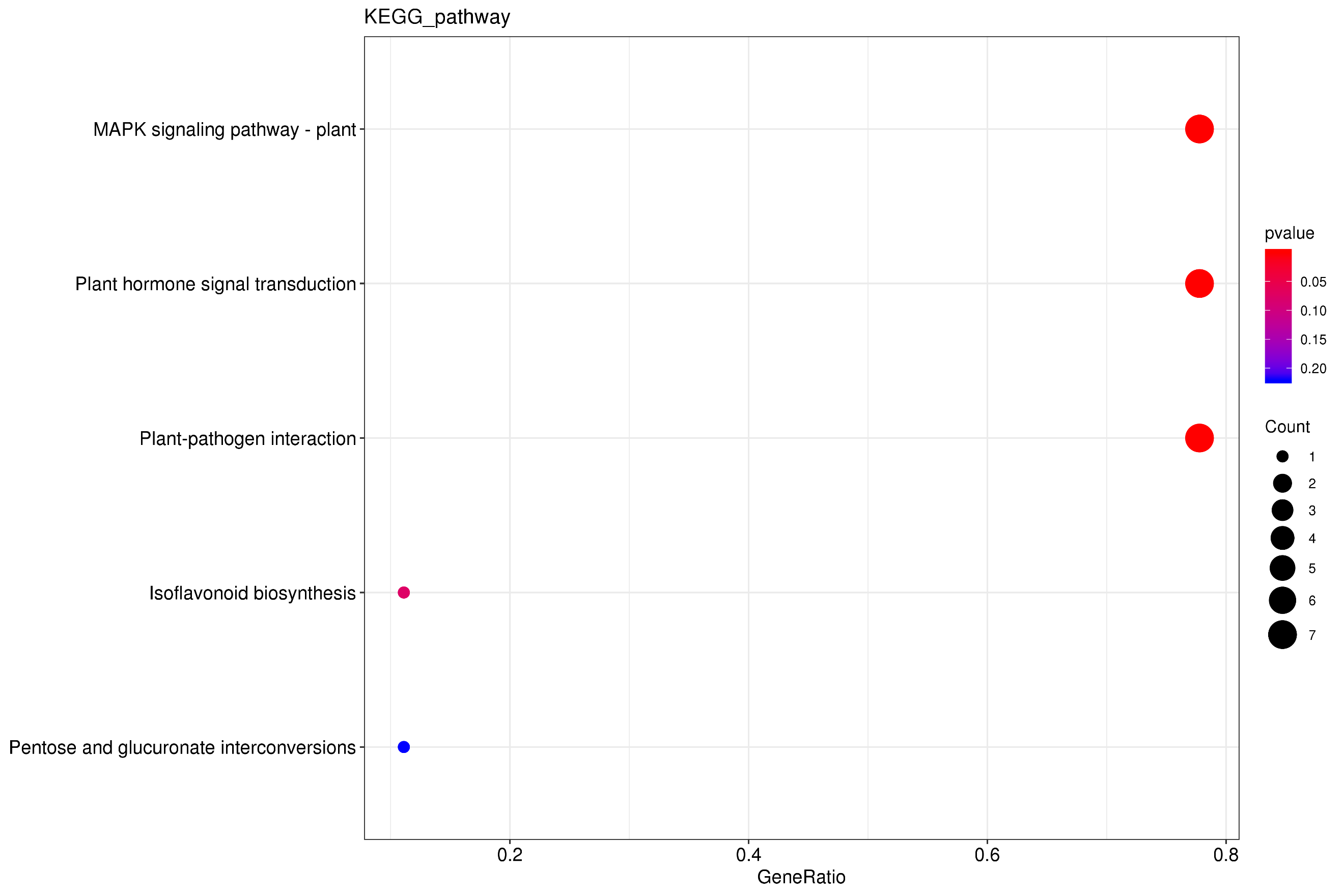

Supplement: Supplementary Figure 1 — GO and KEGG analysis of target genes of DElncRNAs. (A,B) GO analysis of cis target genes of DElncRNAs. Top 20 terms of biological process (A) and molecular function (B) were displayed. (C,D) GO analysis of trans target genes of DElncRNAs. Top 20 terms of biological process (C) and molecular function (D) were displayed. (E) KEGG analysis of cis target genes of DElncRNAs. Top 20 pathways were displayed (F) KEGG analysis of trans target genes of DElncRNAs. Top 20 pathways were displayed. [file Data_Sheet_1.docx]
